# Synthesis and Characterisation of Hemihydrate Gypsum–Polyacrylamide Composite: A Novel Inorganic/Organic Cementitious Material

**DOI:** 10.3390/ma17071510

**Published:** 2024-03-26

**Authors:** Yuan Chen, Zerui Mi, Jiatong Yang, Xuan Zheng, Huihu Wang, Marie-Christine Record, Pascal Boulet, Juan Wang, Jan-Michael Albina, Yiwan Huang

**Affiliations:** 1 Hubei Provincial Key Laboratory of Green Materials for Light Industry, Collaborative Innovation Center of Green Light-Weight Materials and Processing, and School of Materials and Chemical Engineering, Hubei University of Technology, Wuhan 430068, China; zeruimi72@gmail.com (Z.M.); yangjiatong554@gmail.com (J.Y.); zx88@hbut.edu.cn (X.Z.); wanghuihu@hbut.edu.cn (H.W.); jwang@hbut.edu.cn (J.W.); albina@hbut.edu.cn (J.-M.A.); 2 New Materials and Green Manufacturing Talent Introduction and Innovation Demonstration Base, Wuhan 430068, China; m-c.record@univ-amu.fr (M.-C.R.); pascal.boulet@univ-amu.fr (P.B.); 3 Aix-Marseille University, IM2NP, 13397 Marseille, CEDEX 20, France; 4 CNRS, IM2NP, 13397 Marseille, CEDEX 20, France

**Keywords:** gypsum–polymer composite, polyacrylamide hydrogel, dual network structure, mechanical enhancement, initial setting time

## Abstract

This study combined inorganic α-hemihydrate gypsum (*α*-HHG) with organic polyacrylamide (PAM) hydrogel to create a novel *α*-HHG/PAM composite material. Through this facile composite strategy, this fabricated material exhibited a significantly longer initial setting time and higher mechanical strength compared to *α*-HHG. The effects of the addition amount and the concentration of PAM precursor solution on the flowability of the *α*-HHG/PAM composite material slurry, initial setting time, and mechanical properties of the hardened specimens were investigated. The structural characteristics of the composite material were examined using XRD, FE-SEM, and TGA. The results showed that the initial setting time of the *α*-HHG/PAM composite material was 25.7 min, which is an extension of 127.43% compared to that of *α*-HHG. The flexural strength and compressive strength of the oven-dried specimens were 23.4 MPa and 58.6 MPa, respectively, representing increases of 34.73% and 84.86% over values for *α*-HHG. The XRD, FE-SEM, and TGA results all indicated that the hydration of *α*-HHG in the composite material was incomplete. The incompleteness is caused by the competition between the hydration process of inorganic *α*-HHG and the gelation process of the acrylamide molecules for water, which hinders some *α*-HHG from entirely reacting with water. The enhanced mechanical strength of the *α*-HHG/PAM composite material results from the tight interweaving and integrating of organic and inorganic networks. This study provides a concise and efficient approach to the modification research of hemihydrate gypsum.

## 1. Introduction

Gypsum, a traditional inorganic cementitious material, has found extensive application across various sectors including architectural ornamentation, craft arts, precision casting, and as functional fillers [1,2]. It exists in three forms: dihydrate, hemihydrate (subdivided into *α* and *β* types), and anhydrite (subdivided into types I, II, and III). Among these, *α*-type hemihydrate gypsum (*α*-HHG) stands out for practical use, owing to its superior crystal morphology, coarser grain structure, minimal specific surface area, and formation of a stronger, denser, hydration-hardening body upon water addition [3,4]. With the expansion of industries such as self-leveling materials, construction boards, and precision casting, there has been a surge in the demand for enhanced mechanical strength, reduced initial setting time, and improved performance characteristics of *α*-HHG in engineering applications [5,6,7].

To tailor the initial setting time and bolster the mechanical strength of gypsum materials, significant research has focused on their modification, such as the incorporation of organic polymers and inorganic additives into gypsum systems [8,9,10,11,12,13]. Yu et al. notably enhanced the toughness and strength of composites by integrating organic and inorganic units at a molecular scale into an inorganic base [14]. Mroz et al. applied hydroxyethyl methyl cellulose to alter the morphology of calcium sulphate dihydrate crystals, thereby improving the material’s flexural strength [15]. Ding et al. harnessed the degradation products of leather to create hydrogels that, when mixed with gypsum, produced a lightweight variant aimed at boosting gypsum’s thermal insulation properties [16]. Thompson et al. employed hydrogel beads and methyl cellulose to control the porosity and pore size distribution in gypsum through templating, enhancing both its water resistance and sound insulation capabilities [17]. Pedrajas et al. combined concentrated slurries containing polystyrene nano-particles (NPSs) with gypsum to fabricate lightweight gypsum composite materials, demonstrating exceptional thermal insulation and waterproofing properties [18]. Charai et al. utilised coal fly ash (CFA) as a porogen and plant fibres as both a moisture absorber and a reinforcing agent to produce gypsum-based lightweight composite materials. These materials exhibit good thermal insulation and moisture absorption capabilities, with potential applications in the thermal and humidity regulation of buildings [19]. Despite these advances, achieving a balance between enhanced mechanical properties and optimal initial setting time remains a challenge.

Polymer hydrogels are crosslinked hydrophilic networks that are capable of swelling in water and retaining substantial quantities of water without dissolution [20,21]. Polyacrylamide (PAM) hydrogels represent a significant subset of synthetic polymer hydrogels, known for their straightforward synthesis and the abundance of amide groups within their structure. These groups are conducive to forming hydrogen bonds, endowing the hydrogels with excellent water absorption and stability [22,23,24,25,26,27]. We hypothesise that integrating *α*-HHG with PAM hydrogel could yield a dual network structure. This structure would combine an inorganic gypsum base network with an organic polymer hydrogel network, potentially enhancing the mechanical characteristics of gypsum materials and prolonging the initial setting time of *α*-HHG by utilising the water retention capabilities of the polymer hydrogel.

The present investigation involved the preparation of *α*-HHG-based polyacrylamide hydrogel composites (*α*-HHG/PAM) in accordance with the aforementioned ideas. This resulted in the realisation of an effective composite of an organic polymer network and an inorganic gypsum network, markedly enhanced the coagulation properties of *α*-HHG materials, and significantly improved the mechanical properties of the composites. This approach is concise and efficient, providing a fresh perspective on the modification research of hemihydrate gypsum. Moreover, this study offers an approach to augment the efficacy of gypsum resource utilisation [28].

## 2. Materials and Methods

### 2.1. Materials

The raw materials consisted of *α*-HHG (*α*-type gypsum hemihydrate, CaSO_4_·0.5H_2_O, powder, Dinglijie Gypsum Powder Co., Ltd., Zaozhuang, China, water requirement for standard consistency of 36 g (per 100 g *α*-HHG), specific surface area of 0.4 m^2^/g), AM (acrylamide, C_3_H_5_NO, Shanghai McLean Biochemicals Co., Ltd., Shanghai, China, AR), APS (ammonium persulphate, (NH_4_)_2_S_2_O_8_, Shanghai McLean Biochemicals Co., Ltd., Shanghai, China, AR), MBA (N′N-methylenebisacrylamide, C_7_H_10_N_2_O_2_, Shanghai McLean Biochemicals Co. Ltd., Shanghai, China, AR) and TEMED (tetramethylethylenediamine, C_6_H_16_N_2_, Aladdin Reagent Co., Ltd., Shanghai, China, AR). Solutions were prepared with deionised water (Molecular Lab water ultra-purifier, Shanghai, China).

### 2.2. Preparation of PAM Precursor Solutions

Table 1 shows the composition of the three PAM precursor solutions. As an example, PAM-C2.5 was prepared by dissolving 2.5 mol/L AM, 0.0025 mol/L APS (as an initiator), and 0.0025 mol/L MBA (as a crosslinker) in deionised water at room temperature and stirring until complete dissolution, followed by adding 0.0086 mol/L TEMED (as an accelerator) to the solution and stirring well.

### 2.3. Preparation and Mechanical Property Testing of α-HHG/PAM Composites

**Figure 1:**
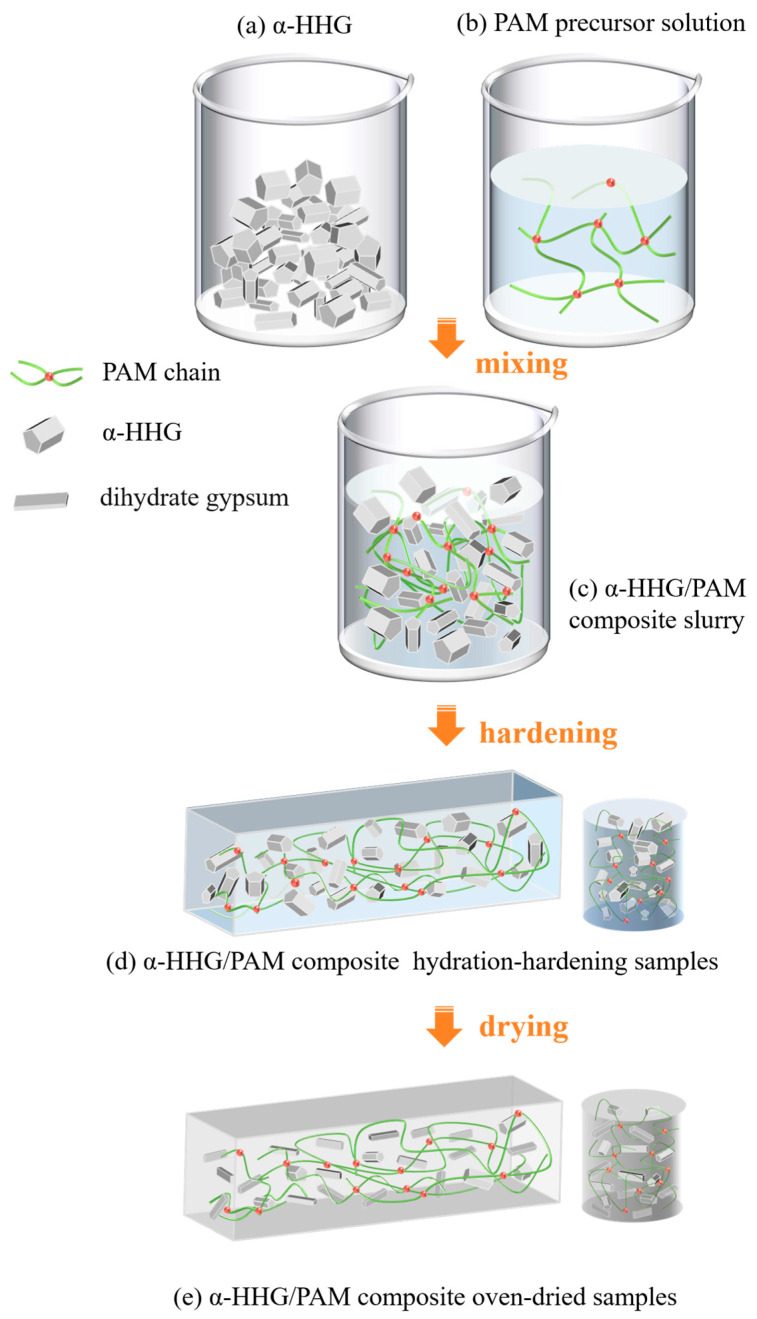
Schematic diagram of the preparation process for the α-HHG/PAM composite material.

Figure 1 illustrates the process for synthesising *α*-HHG/PAM composites. The above PAM precursor solutions were mixed (immediately after the preparation) with *α*-HHG in a certain proportion and stirred well to produce the α-HHG/PAM composite slurry (as shown in Figure 1c). The slurry was poured into moulds and left to undergo hydration and hardening at room temperature (20 ± 3 °C) to produce the *α*-HHG/PAM composite hydration-hardening specimens (Figure 1d). These specimens were then dried at 40 °C in a drying oven until a constant weight was reached, resulting in the *α*-HHG/PAM composite oven-dried specimens (Figure 1e).

With reference to GB/T (17669.3-1999) [29] “Gypsum plasters: Determination of mechanical properties”, the flexural and compressive strengths of the hydration-hardening specimens and the oven-dried specimens of the aforementioned *α*-HHG/PAM composites were tested using the YAW-300C Mechanical Testing Machine (Jinan Zhongluchang Testing Machine Manufacturing Co., Ltd., Jinan, China). Flexural strength was tested using a rectangular specimen (60 × 20 × 20 mm) loaded at a rate of 20 N/s, while compressive strength was tested using a cylindrical specimen (Φ20 × 20 mm) loaded at a rate of 2000 N/s. The same specimen was tested three times, and the average value was taken as the value of its mechanical strength.

### 2.4. Evaluation of Physical Properties of α-HHG/PAM Composite Slurry

The physical properties of *α*-HHG/PAM composite slurry were characterised in terms of three factors: temperature change, initial setting time, and flowability. The test methods refer to GB/T(17669.5-1999) [30] “Gypsum plasters: Determination of physical properties of powder”.

(1) *Temperature Change.* PAM precursor solution was added to 100 g of α-HHG and thoroughly mixed to formulate a standard-consistency *α*-HHG/PAM composite slurry. Subsequently, an automated temperature sensor (DS18B20, Dallas Semiconductor, Dallas, TX, USA) was promptly inserted into the slurry. The temperature changes during the hydration and hardening of the slurry were tested at room temperature (20 °C ± 3 °C).
(2) *Initial Setting Time.* The initial setting time of *α*-HHG/PAM composites was determined by using a Vicat apparatus (ISO, Wuxi Zhongke Building Material Instrument Co., Ltd., Wuxi, China). As shown in Figure S1, the *α*-HHG/PAM composite slurry of the above standard consistency was poured into the annular mould of the Vicat apparatus, and the time was counted from when the *α*-HHG was mixed with the PAM precursor solution to the time when the Vicat apparatus pointer exceeded 1 and was stopped (measured at intervals of 30 s), which is the initial solidification time of the *α*-HHG/PAM composite.
(3) *Flowability.* The flowability of the *α*-HHG/PAM composite slurry was determined using the diameter measurement method as shown in Figure S2. The α-HHG/PAM composite slurry of the above standard consistency was poured into the standard mould (LT-JJLD, Beijing Zhongke Luda Test Instrument Co., Ltd., Beijing, China) as soon as it was prepared, making sure the liquid level of the slurry was flush with the top. The mould was then rapidly lifted vertically to allow the slurry to flow naturally. After 15 s, the diameter of the slurry on the glass plate was measured, and the value was used to indicate the fluidity of the slurry.

### 2.5. Structural Analysis of α-HHG/PAM Composites

The *α*-HHG/PAM composite oven-dried specimens were ground and sieved through a 200-mesh sieve, and the structural properties of the powders were characterised by X-ray diffraction (XRD) using an X-ray diffractometer (PANalytical, Empyrean, Almelo, The Netherlands) with Cu Kα radiation (λ = 0.154 nm) and a 2θ scan range of 20~90° at a rate of 2°/min. 

Field emission scanning electron microscopy (FE-SEM, SU8010, Hitachi, Tokyo, Japan) operating at 5 kV was used to observe the microscopic morphology of α-HHG raw material powder and the fracture of hydration-hardening specimens of *α*-HHG/PAM composites. Before fracture observation, the fractured specimens were freeze-dried for 12 h and dried at 40 °C for 12 h.

### 2.6. Thermogravimetric Analysis

Thermogravimetric (TG) analysis was conducted to examine the thermal behaviour of the specimens utilising a simultaneous thermal analysis apparatus (SDT Q600, TA Instruments, New Castle, DE, USA). This was performed under an inert argon atmosphere (flow rate of 40 mL/min) from ambient temperature up to 300 °C at a heating rate of 10 °C/min.

### 2.7. Water Resistance Analysis of α-HHG/PAM Composites

Six cylindrical specimens of *α*-HHG/PAM composites were made using the method described above for compressive strength testing. All specimens were then dried in a drying oven at 40 °C until they reached a consistent weight. Three of them underwent direct compressive strength testing, and the average value obtained was used as the dry compressive strength of specimen *R*1. The remaining three specimens were immersed in water for 24 h, and the average value obtained from these tests represents the wet compressive strength of specimen *R*2. The softening coefficient *f* of the composite material can be determined by calculating the ratio of *R*2 to *R*1, as indicated in Equation (1).

*f* = *R*2/*R*1 (1)

## 3. Results and Discussion

### 3.1. Influence of PAM Precursor Addition on the Properties of α-HHG/PAM Composites

#### 3.1.1. Influence on the Flowability of α-HHG/PAM Composite Slurries

**Figure 2:**
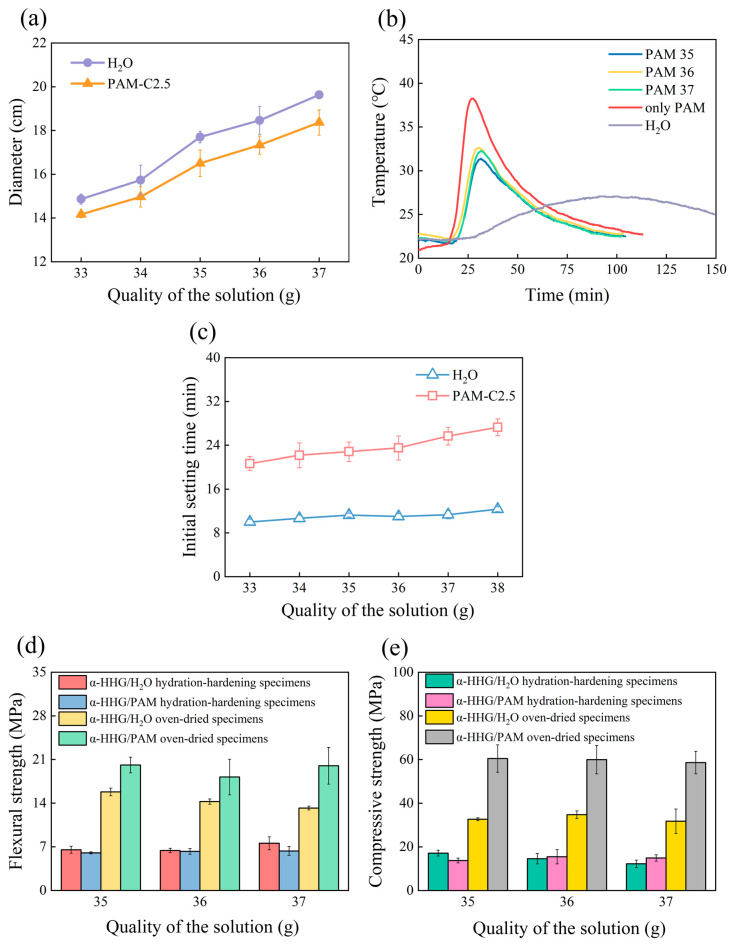
Variation in the properties of α-HHG/PAM-C2.5 composite material with the addition of PAM precursor solution: (**a**) flowability, (**b**) hydration-hardening temperature, (**c**) initial setting time, (**d**) flexural strength, and (**e**) compressive strength. The horizontal axis, labelled “Quality of the solution”, indicates the mass of solution added per 100 g of α-HHG. The curve labelled “H_2_O” represents the α-HHG control group with only pure water added (i.e., α-HHG/H_2_O), whereas the curve labelled “PAM-C2.5” denotes the 2.5 mol/L PAM hydrogel without added α-HHG.

Due to the significant impact of the consistency of gypsum slurry on the workability of gypsum materials in practical engineering, when studying the effect of the addition amount of PAM precursor liquid on the flowability of *α*-HHG/PAM composite slurry, we only vary the consistency of the *α*-HHG/PAM composite slurry around the standard consistency of the *α*-HHG slurry. The flowability of the *α*-HHG/PAM composite slurry as a function of the addition of PAM-C2.5 precursor liquid is shown by the orange curve in Figure 2a. The purple curve in the same figure represents the control group where pure water is added solely to *α*-HHG. From the figure, it can be observed that at the same addition level, the flowability of the *α*-HHG/PAM composite slurry is lower compared to that of the control group where pure water is added. This is attributed to the higher viscosity of the hydrophilic PAM network compared to that of pure water. Regarding the variation in the addition level, as the amount of PAM precursor liquid increases, the flowability of the *α*-HHG/PAM composite slurry continuously increases. This is due to the increased proportion of the liquid phase in the slurry. The standard-consistency water requirement for *α*-HHG used in this study is 36 g, meaning that the slurry reaches the standard consistency when 36 g of pure water is added to every 100 g of *α*-HHG. From the figure, it can be observed that when the addition level of PAM precursor liquid is 37 g, the consistency of the *α*-HHG/PAM composite slurry is closest to the standard consistency of *α*-HHG slurry.

#### 3.1.2. Influence on the Hydration-Hardening Temperature of α-HHG/PAM Composites

Figure 2b presents the variation curves of hydration-hardening temperature over time for *α*-HHG/PAM composite materials with different addition levels of PAM precursor liquid. PAM 35, PAM 36, and PAM 37 represent the addition levels of 35 g, 36 g, and 37 g of PAM-C2.5 precursor liquid per 100 g of *α*-HHG, respectively. The curve labelled “H_2_O” indicates the case where only pure water is added to *α*-HHG, and hydration-hardening is conducted under standard consistency. The curve labelled “only PAM” represents the scenario where only PAM-C2.5 precursor liquid is present without *α*-HHG. 

From the figure, it can be observed that the temperature of each system exhibits an initial increase followed by a subsequent decrease during the reaction process. When *α*-HHG is mixed only with pure water and subjected to hydration hardening under standard-consistency conditions, the rate of temperature increase in the system is relatively slow. It reaches its peak temperature (approximately 27 °C) after 98 min and then gradually decreases. When the system contains only PAM-C2.5 precursor solution, the temperature slowly rises from 21 °C to 23 °C within the first 16 min. Subsequently, due to its own gelation reaction, the temperature rapidly increases, reaching its peak temperature (approximately 38 °C) at 27 min, after which it begins to decrease. The initial temperatures of the PAM35, PAM36, and PAM37 systems are all around 23 °C. They experience a slight decrease of 1–2 °C in the first 18 min, followed by a rapid increase. Around 30 min, they reach their peak temperatures (approximately 32 °C) before beginning to decrease. It can also be observed that variations in the addition of PAM-C2.5 precursor solution have a minimal impact on the hydration-hardening temperature of the *α*-HHG/PAM composite materials.

Compared to the pure PAM-C2.5 precursor solution group (red curve), the maximum reaction temperature of the *α*-HHG/PAM composite material decreases, and the time to reach the peak temperature is delayed. This indicates that the presence of *α*-HHG retards the gelation reaction of the acrylamide solution. Since water activity is one of the key factors controlling the gelation rate of acrylamide, and both the gelation of acrylamide and the hydration hardening of *α*-HHG require water [31,32,33,34], the aforementioned phenomenon suggests that a competitive situation for water may arise between the gelation process of organic acrylamide solution and the hydration-hardening process of inorganic *α*-HHG in the *α*-HHG/PAM system. This competition for water delays the two aforementioned reactions.

#### 3.1.3. Influence on the Initial Setting Time of α-HHG/PAM Composites

Figure 2c illustrates the variation curve of the initial setting time of the system when different masses of pure water or PAM-C2.5 precursor solution are added to 100 g of α-HHG. From the figure, it is evident that compared to the pure water group, the initial setting time of the α-HHG/PAM composite material significantly increases. Moreover, the initial setting time of the α-HHG/PAM composite material extends with an increase in the amount of PAM-C2.5 precursor solution added. When the PAM precursor solution’s addition reaches 37 g, the initial setting time is the longest, reaching 25.7 min, representing a 127.43% increase compared to the pure water group’s 11.3 min. The increase in initial setting time indicates a delay in the hydration-hardening reaction of α-HHG, further confirming the speculation made in Section 3.1.2. In the composite material system, the hydration-hardening process of inorganic α-HHG competes with the gelation process of organic acrylamide solution for water, thus slowing down both reactions. The significant extension of the initial setting time in practical engineering will provide the α-HHG/PAM composite material slurry with a longer period for flow, shaping, and manipulation. This holds great practical engineering significance.

#### 3.1.4. Influence on the Mechanical Properties of α-HHG/PAM Composites

The variation in flexural strength of hydration-hardening and oven-dried specimens for both the pure water system (*α*-HHG/H_2_O) and the PAM-C2.5 system (*α*-HHG/PAM) with changes in the amount of water or PAM-C2.5 added is depicted in Figure 2d. From the figure, it can be observed that for the hydration-hardening specimens, there is little difference in flexural strength between the pure water system and the PAM-C2.5 system. This phenomenon may be attributed to two factors. Firstly, because the strength testing occurs 24 h after material preparation, in the *α*-HHG/PAM composite material system, the hydration-hardening process of inorganic *α*-HHG and the gelation process of organic acrylamide solution are both delayed due to competition for water. Consequently, neither of these reactions is fully completed by 24 h after preparation. This is further confirmed in the subsequent section on strength kinetics. At this point, the strength advantage of the *α*-HHG/PAM composite material system is not fully demonstrated. Secondly, due to the water retention property of polyacrylamide hydrogels, the water content in the *α*-HHG/PAM composite material system is higher compared to that in the pure water system. At this stage, the interpenetrating double network between organic PAM hydrogel and inorganic *α*-HHG is insufficient and not tightly bonded, resulting in the inability of the composite material to fully exploit its strength advantage.

However, from Figure 2d, it can be observed that for the oven-dried specimens, the flexural strength of the PAM-C2.5 system is significantly higher than that of the pure water system. When the addition amounts are both 37 g, the former is increased by 34.73% compared to the latter. This phenomenon may be attributed to two factors. Firstly, in the PAM-C2.5 oven-dried system, due to sufficient reaction time, both the hydration-hardening process of inorganic *α*-HHG and the gelation process of organic acrylamide solution can proceed adequately. This results in the formation of an intertwined and integrated organic/inorganic dual network (as can be observed in subsequent SEM images), allowing the strength advantage of the composite material to be realised. Secondly, during the drying process, water evaporates, leading to tighter bonding between the organic hydrogel network and the inorganic hydrogel material network in the *α*-HHG/PAM composite material system. Consequently, this system exhibits higher flexural strength. Furthermore, from Figure 2d, it can be observed that for the PAM-C2.5 system, minor variations in the addition of PAM precursor solution have minimal impact on the flexural strength of the composite material.

The compressive strength of hydrated and oven-dried specimens for both the pure water system (*α*-HHG/H_2_O) and the PAM-C2.5 system (*α*-HHG/PAM) with changes in the amount of water or PAM-C2.5 added is illustrated in Figure 2e. Similar to the information displayed for flexural strength, the compressive strength of hydration-hardening specimens of the *α*-HHG/PAM composite material is comparable to that of the pure water system. However, interestingly, there is a more significant increase in the compressive strength of the oven-dried specimens. When the addition of PAM precursor solution is 37 g, the compressive strength of the oven-dried specimens increases by 84.86% compared to the pure water group. Furthermore, minor variations in the addition of PAM precursor solution have a minimal impact on the compressive strength of the material. The reason for this phenomenon is similar to that observed in the flexural scenario, where within the *α*-HHG/PAM composite material system, the interpenetration and bonding of organic and inorganic hydrogel materials’ dual networks significantly enhance the compressive strength of the material. Additionally, this network structure may provide more effective resistance against externally applied compressive stress.

In summary, when 37 g of PAM precursor solution is added to every 100 g of *α*-HHG, the resulting *α*-HHG/PAM composite material slurry approaches standard consistency. The initial setting time increases by 127.43% compared to the pure water system. The strength of the hydration-hardening specimens of the composite material is comparable to that of the pure water system, but the strength of the oven-dried specimens of the composite material is significantly increased. Compared to the pure water group, the flexural strength of the α-HHG/PAM composite material’s oven-dried specimens increases by 34.73%, while the compressive strength increases by 84.86%.

### 3.2. Influence of PAM Precursor Solution Concentration on the Properties of α-HHG/PAM Composites

This section investigates the influence of PAM precursor solution at different concentrations on the properties of *α*-HHG/PAM composite materials. Based on the results of Section 3.1, the mass of PAM precursor solution added was determined to be 37 g per 100 g of *α*-HHG in order to achieve a consistency of the *α*-HHG/PAM composite material slurry close to the standard consistency.

#### 3.2.1. Influence on the Flowability of α-HHG/PAM Composite Slurries

**Figure 3:**
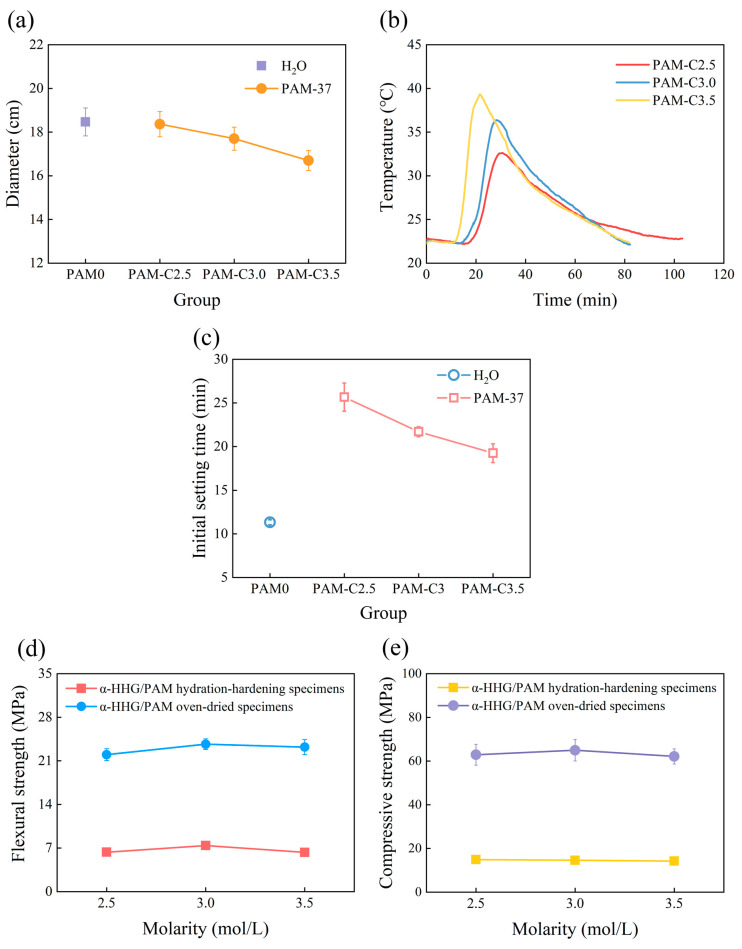
Variation in the properties of α-HHG/PAM37 composite material with the molar concentration of PAM precursor solution: (**a**) flowability, (**b**) hydration-hardening temperature, (**c**) initial setting time, (**d**) flexural strength, and (**e**) compressive strength. “PAM37” denotes the addition of 37 g of PAM precursor solution with varying molar concentrations to every 100 g of α-HHG.

Figure 3a shows the variation curve of the flowability of α-HHG/PAM composite material slurry with the molar concentration of PAM precursor solution, where PAM0 represents the control group with only pure water added to *α*-HHG. As shown in the figure, the flowability of the slurry in the pure water group is 18.46 cm. With the increase in the molar concentration of PAM precursor solution, the flowability of the *α*-HHG/PAM composite material slurry slightly decreases. When the molar concentration of PAM precursor solution is 2.5, 3.0, and 3.5 mol/L, the flowability of the *α*-HHG/PAM composite material slurry is 18.36, 17.7, and 16.70 cm, respectively, all lower than that of the pure water group. This phenomenon occurs because the viscosity of the PAM precursor solution increases with its own concentration, leading to a reduction in the flowability of the *α*-HHG/PAM composite material slurry.

#### 3.2.2. Influence on the Hydration-Hardening Temperature of α-HHG/PAM Composites

Figure 3b illustrates the variation curve of the hydration-hardening temperature of *α*-HHG/PAM composite material with the concentration of PAM precursor solution. *α*-HHG/PAM-C2.5 reached its peak temperature of 33 °C at 29 min, *α*-HHG/PAM-C3.0 reached its peak temperature of 36 °C at 27 min, and *α*-HHG/PAM-C3.5 reached its peak temperature of 39 °C at 21 min. This indicates that with the increase in PAM precursor concentration, the peak temperature during the hydration-hardening process of *α*-HHG/PAM composite materials rises, while the time to reach peak temperature decreases. The underlying cause of this phenomenon is the accelerated reaction rate of organic network gelation due to the higher concentration of PAM precursor solution.

#### 3.2.3. Influence on the Initial Setting Time of α-HHG/PAM Composites

Figure 3c displays the variation in the initial setting time of *α*-HHG/PAM composite materials with the concentration of PAM precursor solution. It can be observed that as the concentration of PAM precursor solution increases, the initial setting time of *α*-HHG/PAM composite materials continuously decreases. The initial setting times of *α*-HHG/PAM composite material slurries are 25, 21, and 19 min when the molar concentrations of PAM precursor solution are 2.5, 3.0, and 3.5 mol/L, respectively. These periods are considerably longer than the 11 min observed for the pure water group. The decrease in initial setting time with increasing PAM precursor solution concentration may be attributed to the following two aspects. Firstly, as depicted in Figure 3b, a higher concentration of PAM precursor solution leads to a faster gelation of the organic polyacrylamide network, resulting in a rapid decrease in system fluidity and hence a shortened initial setting time. Secondly, as illustrated in Figure 3b, a higher concentration of PAM precursor solution results in greater heat release in the system, which accelerates the hydration-hardening reaction of *α*-HHG, further shortening the initial setting time. In any case, due to the presence of organic gel, the competition between the gelation process and the hydration-hardening process of *α*-HHG for water delays the hardening process of the composite system. This results in an overall significantly longer initial setting time for *α*-HHG/PAM composite materials compared to that of the pure water group.

#### 3.2.4. Influence on the Mechanical Properties of α-HHG/PAM Composites

The flexural strength of both the hydration-hardening and oven-dried specimens of *α*-HHG/PAM composite materials, as a function of PAM precursor solution concentration, is depicted in Figure 3d. It can be observed from the figure that the flexural strength of both types of specimens exhibits an initial increase followed by a slight decrease with increasing PAM precursor solution concentration. When the molar concentration of PAM precursor solution is 3.0 mol/L, the strength of *α*-HHG/PAM composite materials is relatively high, with flexural strengths of 7.4 MPa and 23.7 MPa for the hydration-hardening and oven-dried specimens, respectively. It is evident that as the concentration of PAM precursor solution increases, the strength of the formed organic gel network also increases. However, with further increases in PAM precursor solution concentration, the increase in its own viscosity makes it difficult for *α*-HHG to disperse in the system. This hinders the complete interweaving and integrating between the organic gel network and the inorganic *α*-HHG network, resulting in the flexural strength of *α*-HHG/PAM composite materials exhibiting an initial increase followed by a slight decrease with increasing PAM precursor solution concentration.

Figure 3e presents the curve of compressive strength versus the concentration of the PAM precursor solution for hydration-hardening and oven-dried specimens of *α*-HHG/PAM composite materials. It can be observed that the trend of compressive strength variation for *α*-HHG/PAM composite materials is similar to that of flexural strength. When the molar concentration of the PAM precursor solution is 3.0 mol/L, the compressive strength of the oven-dried specimens of *α*-HHG/PAM composite materials reaches 65.0 MPa, representing a 3.23% increase compared to that of specimens at a molar concentration of 2.5 mol/L (62.9 MPa). 

However, in practical engineering applications, the performance of materials needs to be considered comprehensively. Comparing the two scenarios with PAM precursor solution concentrations of 2.5 and 3.0 mol/L, although the former exhibits a 3.23% lower compressive strength in the oven-dried state compared to the latter, the former slurry is closer to the standard consistency, and its initial setting time is extended by 18.43% compared to the latter. Additionally, the reduction in the concentration of the PAM precursor solution also implies a decrease in usage cost. Therefore, among the three experimental groups, a concentration of 2.5 mol/L for the PAM precursor solution is deemed the optimal choice.

Integrating the results and analysis from Section 3.1 and Section 3.2, when the molar concentration of the PAM precursor solution is 2.5 mol/L, the flexural strength of the α-HHG/PAM composite material in the absolutely dry state increases by 34.73% compared to that of the α-HHG material, and the initial setting time extends by 127.43%. Both crucial indicators, flexural strength and initial setting time, experience significant positive improvements. This suggests that the α-HHG/PAM composite material possesses a higher service strength. Additionally, in practical engineering, the prolonged initial setting time provides the slurry with a longer period for flow, shaping, and manipulation. Interestingly, the comprehensive cost of the PAM precursor solution is low, and the preparation method for the α-HHG/PAM composite material is relatively simple, requiring only the straightforward mixing of α-HHG with the PAM precursor solution. The aforementioned analysis indicates that the α-HHG/PAM composite material has promising application prospects in practical engineering.

### 3.3. Phase Analysis of α-HHG/PAM Composite Materials

**Figure 4:**
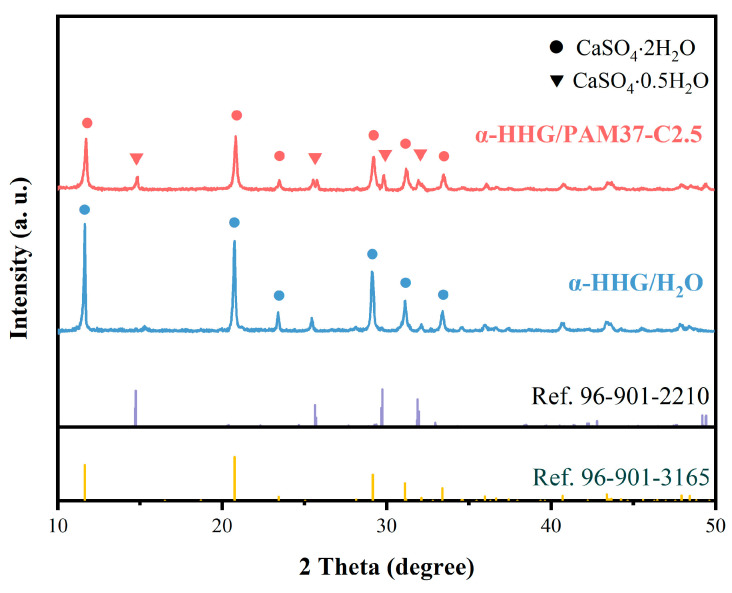
X-ray diffraction patterns of α-HHG/PAM37-C2.5 composite material and α-HHG/H_2_O in hydration-hardened states.

The X-ray diffraction images of the hydration-hardening specimens of *α*-HHG/H_2_O and *α*-HHG/PAM37-C2.5 composite materials are presented in Figure 4. It is evident that the diffraction peaks of the hydration-hardening *α*-HHG/PAM37-C2.5 composite material correspond to those of dihydrate gypsum (CaSO_4_·2H_2_O, Ref.96-901-3165) and hemihydrate gypsum (CaSO_4_·0.5H_2_O, Ref.96-901-2210). However, the diffraction peaks of the specimens with pure water only correspond to those of dihydrate gypsum (CaSO_4_·2H_2_O, Ref.96-901-3165). These results suggest that, in addition to the dihydrate gypsum, unreacted hemihydrate gypsum is also present in the hydration-hardening specimens of the *α*-HHG/PAM composite materials. This verifies the hypothesis about water competition proposed in Section 3.1.1, indicating that the hydration process of inorganic *α*-HHG and the gelation process of the organic acrylamide molecules within the composite system compete for water. Consequently, this results in partial *α*-HHG not reacting fully with water, ultimately leading to the concurrent presence of both dihydrate and hemihydrate gypsum in the *α*-HHG/PAM composite system.

### 3.4. Fracture Analysis of α-HHG/PAM Composite Materials

**Figure 5:**
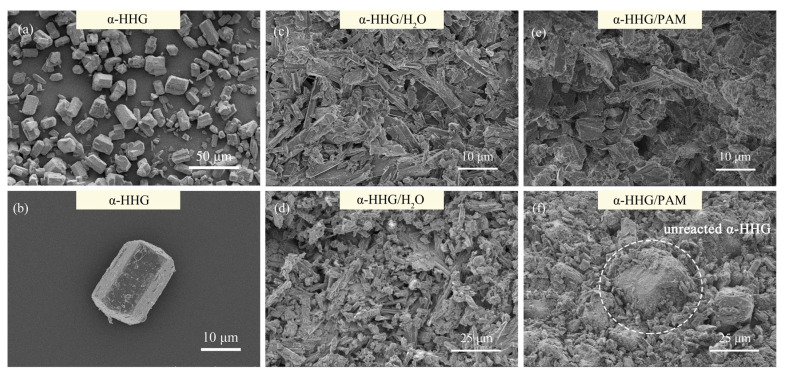
FE-SEM images of (**a**,**b**) raw α-HHG, (**c**,**d**) oven-dried specimen fractures of α-HHG/H_2_O, and (**e**,**f**) oven-dried specimen fractures of α-HHG/PAM37-C2.5 composite material.

Figure 5a,b shows the FE-SEM photographs of the *α*-HHG raw materials, revealing that the *α*-HHG particles predominantly exhibit a hexagonal prism morphology with a particle length-to-diameter ratio of approximately 1.0 to 1.5, demonstrating good monodispersity.

Figure 5c,d displays the FE-SEM photographs of the fracture cross-sections of the oven-dried specimens of *α*-HHG/H_2_O after hydration hardening, showing that the internal crystals are mostly elongated rod-shaped or flake-shaped, with some fine crystals adhering between them. According to the XRD results presented in Figure 4, these crystals are identified as the dihydrate gypsum phase.

Figure 5e,f presents the FE-SEM photographs of the fracture cross-sections of the oven-dried specimens of *α*-HHG/PAM37-C2.5 composite material after hydration hardening. It can be observed that the surfaces of the internal crystals are not as smooth and flat as those in the *α*-HHG/H_2_O specimens, suggesting the presence of the organic polyacrylamide network. Furthermore, the portion marked by the white dashed box in Figure 5f, inferred to be unreacted *α*-HHG particles based on their shape, corroborates the phase analysis results discussed in Section 3.3. This incomplete conversion of *α*-HHG into dihydrate gypsum is attributed to the competition for water between the hydration process of inorganic *α*-HHG and the gelation process of the organic acrylamide solution. The tight interweaving and integrating of the organic and inorganic networks within the *α*-HHG/PAM composite materials significantly enhance the mechanical strength of the composite material.

### 3.5. Thermogravimetric Analysis of α-HHG/PAM Composite Materials

**Figure 6:**
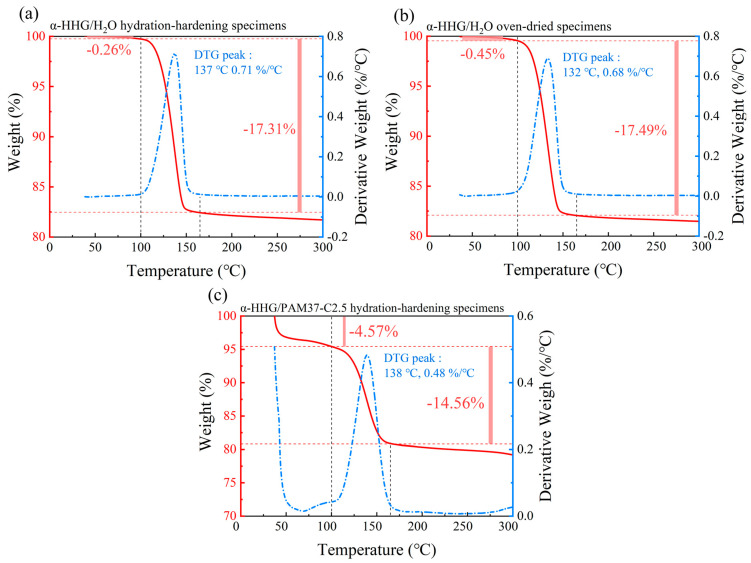
Thermogravimetric analysis (TGA) curves for (**a**) hydration-hardening specimens of α-HHG/H_2_O, (**b**) oven-dried specimens of α-HHG/H_2_O, and (**c**) hydration-hardening specimens of α-HHG/PAM37-C2.5 composite material. The red curve illustrates the proportion of sample mass change with temperature, while the blue curve indicates the dependency of the rate of mass change on temperature, obtained through differentiation of the TGA curve.

The thermogravimetric analysis (TGA) curves for the *α*-HHG/H_2_O control group specimens (prepared to a standard consistency, i.e., 36 g of pure water added per 100 g of *α*-HHG) in both the hydration-hardened and the oven-dried states are depicted in Figure 6a,b, respectively. The TGA curve of the *α*-HHG/PAM37-C2.5 composite material in its hydration-hardened state is shown in Figure 6c. Within Figure 6, the red curve illustrates the proportion of sample mass change with temperature, while the blue curve indicates the dependency of the rate of mass change on temperature, obtained through differentiation of the TGA curve.

As demonstrated in Figure 6a, the hydration-hardening specimens of *α*-HHG/H_2_O in the pure water control group exhibit minimal weight loss before 100 °C, amounting to only 0.26%. The primary phase of weight loss occurs between 100 and 165 °C, with a weight reduction of 17.31%, corresponding to the loss of structural water within the *α*-HHG hydration-hardening specimens [35,36]. The changes in the TGA curve in Figure 6b are similar to those observed in Figure 6a, indicating that the main weight loss (17.49%) of the oven-dried specimens after *α*-HHG hydration-hardening, within the 100–165 °C range, is essentially equivalent to the weight loss of the *α*-HHG hydration-hardening specimens. This suggests that *α*-HHG has completely reacted with the water in the system during the hydration-hardening process.

From Figure 6c, it is evident that the hydration-hardening specimens of *α*-HHG/PAM composite material exhibits significant weight loss before 100 °C, amounting to 4.57%. This reduction in weight may correspond to the portion of water present within the organic gel network of the *α*-HHG/PAM composite material, which might be free in the gel network and leads to system weight loss due to evaporation before reaching 100 °C. Furthermore, Figure 6c reveals that the system undergoes a noticeable weight loss between 100 and 165 °C, with a reduction of 14.56%. As previously mentioned, this weight loss corresponds to the loss of structural water in the *α*-HHG hydration-hardening specimens, a value that is lower than the weight loss observed in the *α*-HHG/H_2_O pure water control group within the same temperature range (17.31%). This suggests that the hydration of *α*-HHG within the *α*-HHG/PAM composite material system is not complete (even though the mass ratio of *α*-HHG in both systems is the same). This is consistent with the SEM images presented in Figure 5f and further corroborates the hypothesis proposed earlier, suggesting a competitive scenario for water usage between the organic gel network and the inorganic *α*-HHG network.

### 3.6. Investigation into the Hydration-Hardening Kinetics of α-HHG/PAM Composite Materials

**Figure 7:**
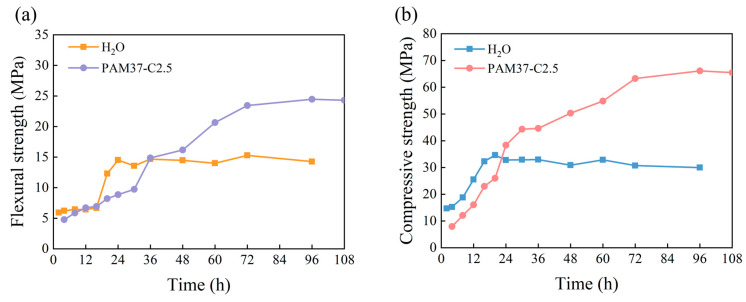
Variation curves of (**a**) flexural strength and (**b**) compressive strength over reaction time for hydration-hardening specimens of α-HHG/PAM37-C2.5 composite material and α-HHG/H_2_O.

The strength variations over time for the hydration-hardening specimens of the *α*-HHG/H_2_O pure water control group (prepared to the standard consistency, i.e., 36 g of pure water added per 100 g of *α*-HHG) and the *α*-HHG/PAM composite material (*α*-HHG/PAM37-C2.5) are shown in Figure 7, where Figure 7a,b respectively depicts the changes in flexural and compressive strength over time.

From Figure 7a, it can be observed that the flexural strength of the specimens in the pure water control group slowly increases during the initial 16 h, rapidly grows between 16 and 24 h, and then essentially stabilises around 15 MPa after 24 h. The flexural strength of the *α*-HHG/PAM composite material increases at a certain rate from 4 to 30 h and then grows at a relatively faster rate from 30 to 72 h before stabilising. The flexural strengths of the specimens at 24 and 72 h are respectively 8.87 and 23.43 MPa. The flexural strength of the *α*-HHG/PAM composite material begins to exceed that of the pure water control group after 36 h.

As shown in Figure 7b, the compressive strength of the specimens in the pure water control group increases at a certain rate during the first 24 h and then stabilises around 32 MPa. The compressive strength of the *α*-HHG/PAM composite material continues to increase from 4 to 72 h, stabilising after 72 h, with the compressive strengths of the specimens at 24 and 72 h being respectively 38.37 and 63.28 MPa. The compressive strength of the *α*-HHG/PAM composite material begins to surpass that of the pure water control group around 24 h.

The results indicate that the hydration-hardening process of the *α*-HHG/PAM composite material is significantly prolonged. At 24 h, the strength of the hydration-hardening specimens of the *α*-HHG/PAM composite material is lower than that of the pure water control group. This is attributed to the competition for water between the hydration process of inorganic *α*-HHG and the gelation process of the organic acrylamide solution, which inhibits the hydration of *α*-HHG, resulting in a lower degree of hydration of *α*-HHG in the *α*-HHG/PAM composite material compared to that of the pure water control group. However, as the reaction time increases, the hydration process of inorganic *α*-HHG and the gelation reaction of organic PAM continue to advance. Moreover, during this process, it is hypothesised that as PAM gelation proceeds, the free water within the organic gel system may escape from the confines of the gel system and continue to participate in the hydration process of *α*-HHG. In summary, with the increase in reaction time, the interweaving and integrating between the organic and inorganic networks within the *α*-HHG/PAM composite material become increasingly tight. The synergistic effect of the two networks continuously strengthens, resulting in a significant enhancement in the mechanical strength of the composite material.

### 3.7. Water Resistance Analysis

**Figure 8:**
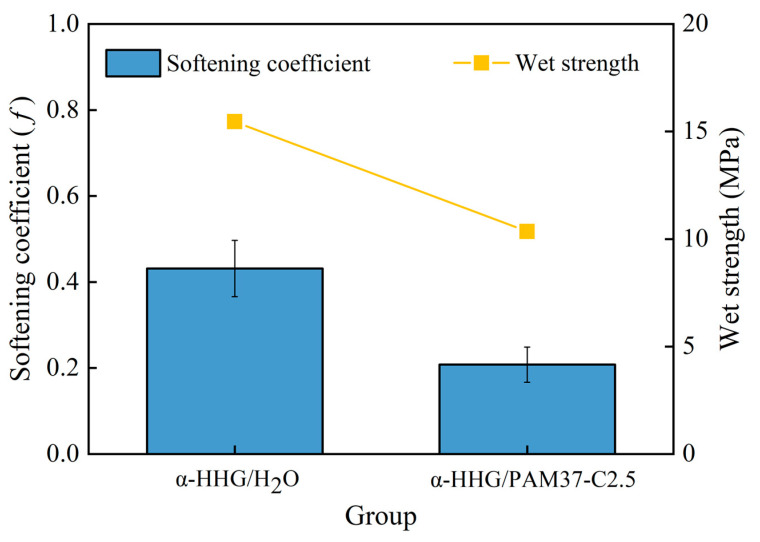
Comparison of softening coefficient (blue bar graph) and wet strength (yellow scatter plots) between α-HHG/H_2_O and α-HHG/PAM37-C2.5 composite materials.

This section evaluates the wet strength (represented by yellow scatter plots) and the softening coefficient *f* (represented by blue bar graph) of the *α*-HHG/H_2_O pure water control group and the *α*-HHG/PAM37-C2.5 composite material, as shown in Figure 8. The softening coefficient reflects the material’s water resistance; a higher softening coefficient indicates better water resistance. The wet strength of the *α*-HHG/H_2_O pure water control group was recorded at 15.5 MPa with a softening coefficient of 0.43. In contrast, the *α*-HHG/PAM37-C2.5 composite material exhibited a wet strength of 10.4 MPa with a softening coefficient of only 0.21. The composite material’s softening coefficient is significantly reduced by 51% compared to that of the pure water control group. This reduction is partly due to the significant increase in the dry compressive strength R1 of the composite material (which grew by 84.86% according to Section 3.1.4) and, on the other hand, the decrease in the wet compressive strength R2 of the composite material by 15.29%, leading to a significant reduction in the ratio of R2/R1, i.e., the softening coefficient. Considering the wet strength alone, the *α*-HHG/PAM37-C2.5 composite material showed a decrease in wet strength after 24 h of water immersion, primarily due to the high water absorbency of the hydrophilic polyacrylamide network within the composite material. In the wet state, the organic hydrogel network absorbs water and swells, while the changes in the already hydrated and hardened inorganic α-HHG are minimal. This may lead to mutual displacement at the interface between the organic hydrogel and inorganic α-HHG, thereby weakening the bond strength between the organic gel network and the inorganic α-HHG network. Additionally, the strength of the organic hydrogel network significantly decreases with water absorption. However, experimental data indicate that the *α*-HHG/PAM37-C2.5 composite material can essentially recover its original absolute dry compressive strength after being dried again after 24 h water immersion. This recovery might be due to the organic hydrogel network regaining its strength upon drying. These findings suggest that the *α*-HHG/PAM37-C2.5 composite material is inferior in water resistance compared to the *α*-HHG/H_2_O pure water control group, imposing certain limitations on its application environments.

## 4. Conclusions

The present study introduces a novel α-HHG/PAM composite material by mixing two materials: inorganic α-hemihydrate gypsum (α-HHG) and organic polyacrylamide (PAM) hydrogel. The effects of the addition and concentration of PAM precursor solution on the fluidity of the α-HHG/PAM composite material slurry, initial setting time, and mechanical properties of hardened specimens were investigated. The XRD, FE-SEM, and TGA techniques were employed to analyse the structural characteristics of the composite material. The results show that compared to the α-HHG pure water control group, the initial setting time of the α-HHG/PAM composite material is significantly extended, and its absolute dry strength is noticeably enhanced. The specific conclusions are as follows:

(i). The initial setting time of the *α*-HHG/PAM composite material is 25.7 min, which is an extension of 127.43% compared to *α*-HHG. The flexural strength and compressive strength of the oven-dried specimens are 23.4 MPa and 58.6 MPa, respectively, representing increases of 34.73% and 84.86% over α-HHG. The enhancement of strength and prolongation of initial setting time make α-HHG/PAM composites more promising for practical engineering applications.
(ii). The strength of α-HHG/PAM composites exhibits minimal variation across different molar concentrations of PAM precursor liquid, specifically 2.5, 3.0, and 3.5 mol/L. However, the initial setting time of the slurry is reduced sequentially, with durations of 25, 21, and 19 min, respectively. Notably, the slurry demonstrates optimal fluidity when the molar concentration of PAM precursor liquid is 2.5 mol/L, resulting in a spreading diameter of 18.36 cm.
(iii). XRD, FE-SEM, and TGA results all indicated that the hydration of *α*-HHG in the composite material was incomplete. The incompleteness is caused by the competition between the hydration process of inorganic *α*-HHG and the gelation process of the organic acrylamide solution for water, which hinders certain *α*-HHG from entirely reacting with water.
(iv). The water resistance of the α-HHG/PAM composite material is inferior to that of the α-HHG/H_2_O pure water control group, with the former having a softening coefficient of 0.21, which is lower than the latter’s 0.43. This imposes certain limitations on its application scenarios.

This study provides a concise and efficient approach to the modification research of hemihydrate gypsum. Currently, research on the toughness of the composite material and the modelling and simulation of its toughening mechanisms are underway.

## Figures and Tables

**Table 1:** Composition of three different PAM precursor solutions.

Precursor Solutions	AM (g)	APS (g)	MBA (g)	TEMED (mL)	Deionised Water (mL)
PAM-C2.5	8.8850	0.0285	0.0193	0.0645	50
PAM-C3.0	10.662	0.0342	0.0231	0.0774	50
PAM-C3.5	12.439	0.0399	0.0270	0.0903	50

## Data Availability

Data are contained within the article and Supplementary Materials.

## Supplementary Materials

The following supporting information can be downloaded at: https://www.mdpi.com/article/10.3390/ma17071510/s1, Figure S1: Schematic diagram of the initial setting time test method for α-HHG/PAM composite material; Figure S2: Schematic diagram of the flowability test method for α-HHG/PAM composite material slurry.

## References

[1] Ravenhill E.R., Kirkman P.M., Unwin P.R. (2016). Microscopic Studies of Calcium Sulfate Crystallization and Transformation at Aqueous-Organic Interfaces. Cryst. Growth Des..

[2] Vidales-Barriguete A., Santa-Cruz-Astorqui J., Piña-Ramírez C., Kosior-Kazberuk M., Kalinowska-Wichrowska K., Atanes-Sánchez E. (2021). Study of the Mechanical and Physical Behavior of Gypsum Boards with Plastic Cable Waste Aggregates and Their Application to Construction Panels. Materials.

[3] Stawski T.M., Besselink R., Chatzipanagis K., Hovelmann J., Benning L.G., Van D., Alexander E.S. (2020). Nucleation pathway of calcium sulfate hemihydrate (bassanite) from solution: Implications for Calcium Sulfates on Mars. J. Phys. Chem. C.

[4] Follner S., Wolter A., Preusser A., Indris S., Silber C., Follner H. (2002). The setting behavior of α- and β- CaSO_4_·0.5H_2_O as a function of crystal structure and morphology. Cryst. Res. Technol..

[5] Li Y., Ni W., Duan P., Zhang S., Wang J. (2022). Experimental Study and Mechanism Analysis of Preparation of α-Calcium Sulfate Hemihydrate from FGD Gypsum with Dynamic Method. Materials.

[6] Barluenga G., Hernandez-Olivares F. (2010). Self-Levelling cement mortar containing grounded slate from quarrying waste. Constr. Build. Mater..

[7] Zając K., Janus M., Morawski A.W. (2019). Improved Self-Cleaning Properties of Photocatalytic Gypsum Plaster Enriched with Glass Fiber. Materials.

[8] Sophia M., Sakthieswaran N. (2019). Synergistic effect of mineral admixture and bio-carbonate fillers on the physico-mechanical properties of gypsum plaster. Constr. Build. Mater..

[9] Dima C., Badanoiu A., Cirstea S., Nicoara A.I., Stoleriu S. (2020). Lightweight Gypsum Materials with Potential Use for Thermal Insulations. Materials.

[10] Wu Q., Zhu Z., Li S., Wang S., Chen B. (2017). Effect of polyacrylic ester emulsion on mechanical properties of macro-defect free desulphurization gypsum plaster. Constr. Build. Mater..

[11] Jia C., Wu L., Chen Q., Ke P., Yoreo D.J.J., Guan B. (2020). Structural evolution of amorphous calcium sulfate nanoparticles into crystalline gypsum phase. Crystengcomm.

[12] Hossain M.S., Ahmed S. (2022). Synthesis of nano-crystallite gypsum and bassanite from waste pila globosa shells: Crystallographic characterization. RSC Adv..

[13] Fantilli A.P., Jóźwiak-Niedźwiedzka D., Denis P. (2021). Bio-Fibres as a Reinforcement of Gypsum Composites. Materials.

[14] Yu Y., Mu Z., Jin B., Liu Z., Tang R. (2020). Organic-inorganic copolymerization for a homogenous composite without an interphase boundary. Angew. Chem.-Int. Edit..

[15] Mroz P., Mucha M. (2018). Hydroxyethyl methyl cellulose as a modifier of gypsum properties. J. Therm. Anal. Calorim..

[16] Ding X., Wang S., Dai R., Chen H., Shan Z. (2022). Hydrogel beads derived from chrome leather scraps for the preparation of lightweight gypsum. Environ. Technol. Innov..

[17] Thompson B.R., Horozov T.S., Stoyanov S.D., Paunov V.N. (2018). Hierarchically porous composites fabricated by hydrogel templating and viscous trapping techniques. Mater. Des..

[18] Pedrajas D.L., Franco M.C., Sáenz I.G., Mellado F.J.R., Romero J.F.R., Simón A.M.B. (2022). Polystyrene nanoparticles slurry as an additive for developing insulating and waterproof gypsum composites. Appl. Therm. Eng..

[19] Charai M., Mghazli M.O., Channouf S., Jagadesh P., Moga L., Mezrhab A. (2023). Lightweight waste-based gypsum composites for building temperature and moisture control using coal fly ash and plant fibers. Constr. Build. Mater..

[20] Bai B., Zhou J., Yin M. (2015). A comprehensive review of polyacrylamide polymer gels for conformance control. Pet. Explor. Dev..

[21] Karoyo A.H., Wilson L.D. (2021). A Review on the Design and Hydration Properties of Natural Polymer-Based Hydrogels. Materials.

[22] Li X., Peng W., Li L., Chen S., Ye L., Peng C. (2022). Simple Synthesis of Copper/MXene/Polyacrylamide Hydrogel Catalyst for 4-nitrophenol Reduction. Mater. Lett..

[23] Spalding B.P., Brooks S.C., Watson D.B. (2010). Hydrogel-encapsulated soil: A tool to measure contaminant attenuation in situ. Environ. Sci. Technol..

[24] Chiang C.Y., Chu C.C. (2015). Synthesis of photoresponsive hybrid alginate hydrogel with photo-controlled release behavior. Carbohydr. Polym..

[25] Murakami K., Aoki H., Nakamura S., Nakamura S., Takikawa M., Hanzawa M., Kishimoto S., Hattori H., Tanaka Y., Kiyosawa T. (2010). Hydrogel blends of chitin/chitosan, fucoidan and alginate as healing-impaired wound dressings. Biomaterials.

[26] Cruz H., Luckman P., Seviour T., Verstraete W., Laycock B., Pikaar I. (2018). Rapid removal of ammonium from domestic wastewater using polymer hydrogels. Sci. Rep..

[27] Sun J.Y., Zhao X., Illeperuma W.R.K., Chaudhuri O., Oh K.H., Mooney D.J., Vlassak J.J., Suo Z. (2012). Highly stretchable and tough hydrogels. Nature.

[28] Haneklaus N., Barbossa S., Basallote M.D., Bertau M., Bilal E., Chajduk E., Chernysh Y., Chubur V., Cruz J., Dziarczykowski K. (2022). Closing the upcoming EU gypsum gap with phosphogypsum. Resour. Conserv. Recycl..

[29] (1999). Gypsum Plasters—Determination of Mechanical Properties.

[30] (1999). Gypsum Plasters—Determination of Physical Properties of Powder.

[31] Nissinen T., Li M., Brielles N., Mann S. (2013). Calcium sulfate hemihydrate-mediated crystallization of gypsum on Ca^2+^-activated cellulose thin films. Crystengcomm.

[32] Zhang J., Lin Z., Wang C., Ma X., Zhang Z., Xiao H. (2023). Stable bassanite bulk phase formed in aqueous solution under the control of polymer-mediated water cctivity. Cryst. Growth Des..

[33] Zhao H., Hu G.H., Ye G.B., Ren X.M., Zhang Q.C., Jiang T. (2018). Effects of superplasticisers on hydration process, structure and properties of α-hemihydrate calcium sulfate. Adv. Cem. Res..

[34] Guan B., Ye Q., Zhang J., Lou W., Wu Z. (2010). Interaction between α-calcium sulfate hemihydrate and superplasticizer from the point of adsorption characteristics, hydration and hardening process. Cem. Concr. Res..

[35] Florian B., João G.D.P., Florian F., Emmanuelle G., Sara Q., Gilles W. (2022). From atom level to macroscopic scale: Structural mechanism of gypsum dehydration. Solid State Sci..

[36] Tang Y.B., Gao J.M., Liu C.B., Chen X.M., Zhao Y.S. (2019). Dehydration pathways of gypsum and the rehydration mechanism of soluble anhydrite γ-CaSO_4_. ACS Omega.

