# Supplementary material for: Synthesis and Characterisation of Hemihydrate Gypsum–Polyacrylamide Composite: A Novel Inorganic/Organic Cementitious Material
Source: Materials (Basel). 2024 Mar 26;17(7):1510. doi: 10.3390/ma17071510 (PMC11012305; doi:10.3390/ma17071510)
Supplement: Supplementary file 1 [file materials-17-01510-s001.zip › materials-2920069-supplementary.pdf]

Supplemental Information

Figure S1. Schematic diagram of the initial setting time test method for α-HHG/PAM composite material.

Figure S2. Schematic diagram of the flowability test method for α-HHG/PAM composite material slurry.

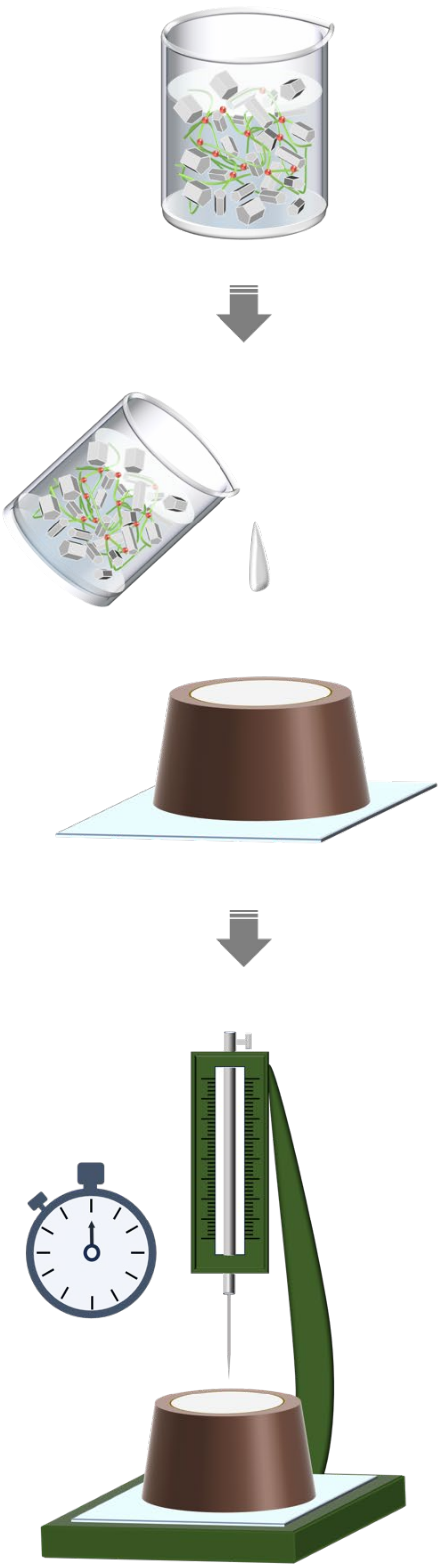

Figure S1. Schematic diagram of the initial setting time test method for α-HHG/PAM composite material.

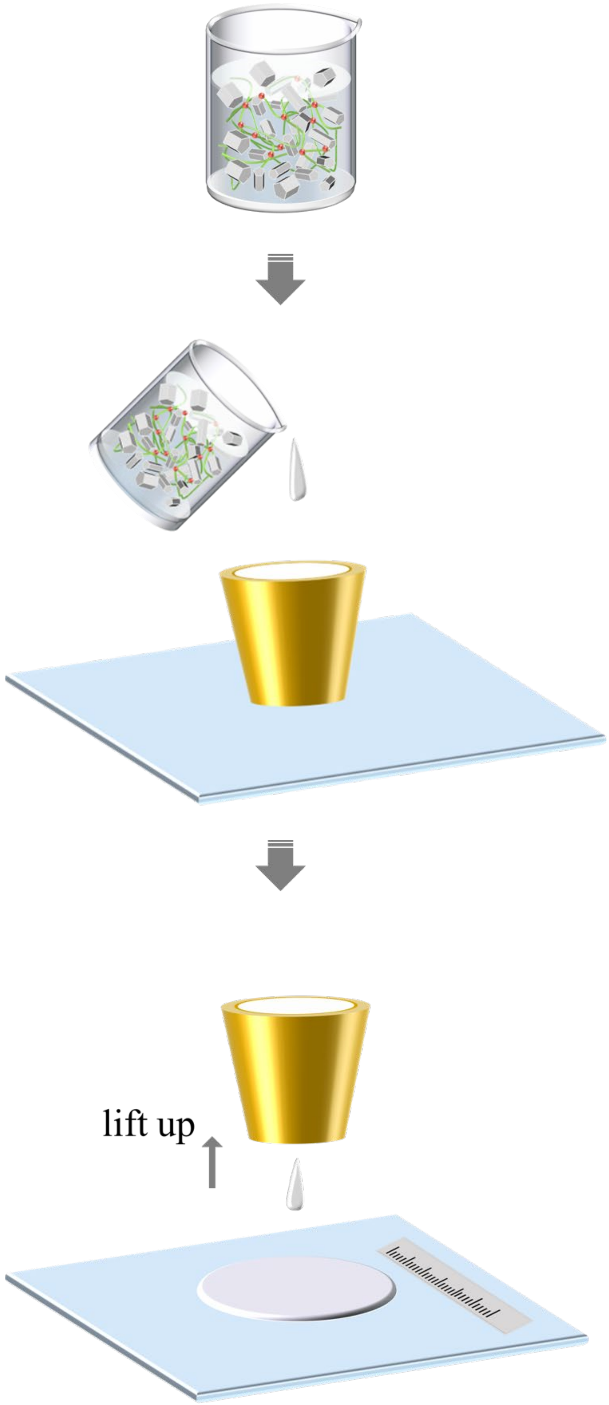

Figure S2. Schematic diagram of the flowability test method for α-HHG/PAM composite material slurry.
